# AMDB: a database of animal gut microbial communities with manually curated metadata

**DOI:** 10.1093/nar/gkab1009

**Published:** 2021-11-08

**Authors:** Junwon Yang, Jonghyun Park, Yeonjae Jung, Jongsik Chun

**Affiliations:** Interdisciplinary Program in Bioinformatics, Seoul National University, Seoul 08826, Korea; Institute of Molecular Biology and Genetics, Seoul National University, Seoul 08826, Korea; Department of Biological Sciences, Seoul National University, Seoul 08826, Korea; Interdisciplinary Program in Bioinformatics, Seoul National University, Seoul 08826, Korea; Institute of Molecular Biology and Genetics, Seoul National University, Seoul 08826, Korea; Department of Biological Sciences, Seoul National University, Seoul 08826, Korea; Interdisciplinary Program in Bioinformatics, Seoul National University, Seoul 08826, Korea; Interdisciplinary Program in Bioinformatics, Seoul National University, Seoul 08826, Korea; Institute of Molecular Biology and Genetics, Seoul National University, Seoul 08826, Korea; Department of Biological Sciences, Seoul National University, Seoul 08826, Korea

## Abstract

Variations in gut microbiota can be explained by animal host characteristics, including host phylogeny and diet. However, there are currently no databases that allow for easy exploration of the relationship between gut microbiota and diverse animal hosts. The Animal Microbiome Database (AMDB) is the first database to provide taxonomic profiles of the gut microbiota in various animal species. AMDB contains 2530 amplicon data from 34 projects with manually curated metadata. The total data represent 467 animal species and contain 10 478 bacterial taxa. This novel database provides information regarding gut microbiota structures and the distribution of gut bacteria in animals, with an easy-to-use interface. Interactive visualizations are also available, enabling effective investigation of the relationship between the gut microbiota and animal hosts. AMDB will contribute to a better understanding of the gut microbiota of animals. AMDB is publicly available without login requirements at http://leb.snu.ac.kr/amdb.

## INTRODUCTION

Animal gut microbiota is a diverse microbial community that lives in the intestine of the host and consists of predominantly bacteria, as well as some archaea, fungi, protozoa and viruses (1). The gut microbiota has received widespread attention due to its potential to influence host physiology (2), immunity (3) and development (4). The gut microbiota has also been hypothesized to contribute to host evolution (5).

The gut microbiota and the host display a bidirectional interaction. Various studies have shown that variations in gut microbiota can be explained by differences in host characteristics (6–8). In particular, host phylogeny and diet largely account for the gut microbiota variations (7). A recent analysis of samples from wild baboons found widespread gut microbiome heritability (9). This vertical transmission may be one of the drivers of phylosymbiosis (10). Phylosymbiosis is defined as ‘microbial community relationships that recapitulate the phylogeny of their host’ (11). Patterns of phylosymbiosis have been reported in many studies (12–14). Additionally, the host diet may also affect the gut microbiota, with several studies reporting that host diet can lead to the convergence of gut microbes in the host species (10,15–18).

Despite the importance of the relationship between gut microbiota and host characteristics, specifically host phylogeny and diet, there is currently no database available that enables easy exploration of the gut microbiota of various animal hosts. Most curated databases focus only on humans (GIMICA (19), GMrepo (20) and HPMCD (21)) and mice (MMDB (22)). There are several databases that contain microbiota data from various animal hosts, including IMNGS (23), MGnify (24), MG-RAST (25) and Qiita (26). However, these databases contain data from various sources other than solely from the animal hosts, making it difficult to identify the relationship between the gut microbiota and animal hosts.

Here, we present Animal Microbiome Database (AMDB) that overcomes these limitations. AMDB includes bacterial 16S ribosomal RNA (rRNA) gene profiles from various animal species to enable the assessment of the relationship between gut microbiota and animal hosts. AMDB currently incorporates 10 478 bacterial taxa and 2530 samples from 34 projects, representing 467 animal species with manually curated metadata. This novel database (i) supports searches by the bacterial taxon of interest, (ii) provides a taxonomic composition of each sample, (iii) incorporates summary information for each project and host and (iv) includes interactive visualizations. Therefore, AMDB will help scientists to quickly access animal gut microbiota data through a user-friendly interface.

## MATERIALS AND METHODS

### Data collection and curation process

**Figure 1.**
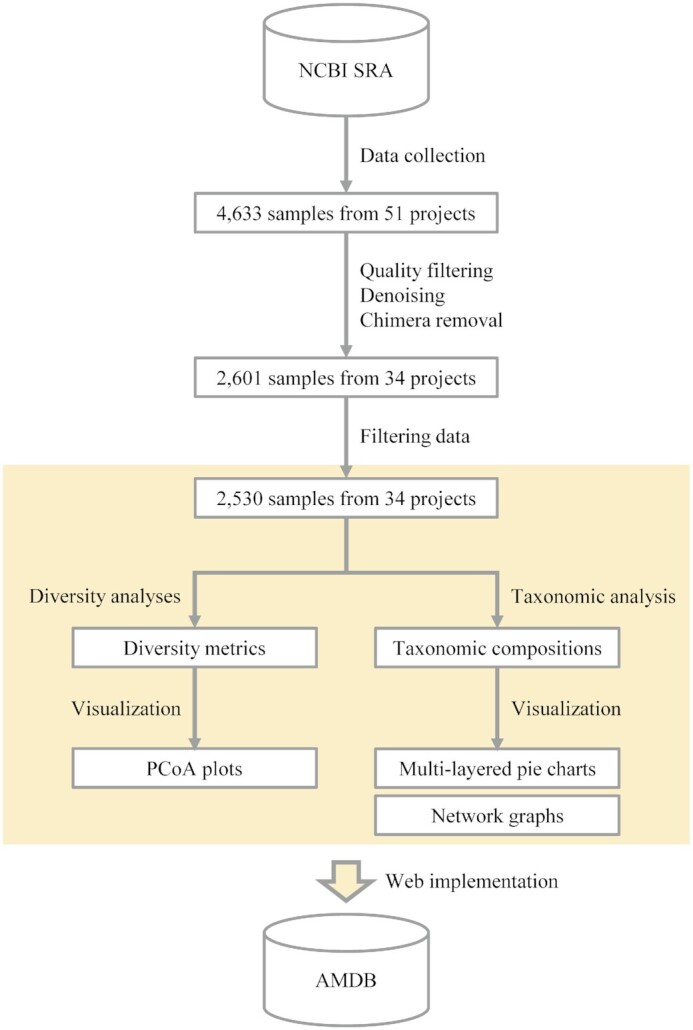
Schematic diagram of AMDB construction. The process name for each step is displayed next to the arrow. The contents contained in AMDB is highlighted in yellow.

We manually selected candidate data for AMDB from the NCBI Sequence Read Archive (SRA) based on the following criteria: (i) samples included fecal or intestinal contents from individual healthy animal hosts, (ii) the PCR primers had to target the V4 hypervariable region of the 16S rRNA gene, (iii) amplicons had to be sequenced on Illumina instruments, (iv) samples had to be linked to research articles. For longitudinal data, only one sample was selected as follows; only one adult sample was included when the samples were from multiple life stages, and one sample from an earlier time point was selected for a given life stage. Samples that were duplicates of those previously included in the AMDB were not included. Amplicon data from different hypervariable regions of the 16S rRNA gene cannot be directly compared due to differences in binding affinity and resolution (27,28). We only used amplicon data from the V4 hypervariable region to ensure comparability. Illumina data was used because we used the Deblur for data processing, which was designed for Illumina data (29). To ensure that samples were of high-quality, we only selected samples linked to research articles. We checked the suitability of samples by reading the publication materials and methods, and we collected metadata, including the accession numbers and the host information. We extracted information on host diets from the MammalDIET (30) and the EltonTraits database (31). A total of 4633 samples were obtained from 51 projects (Figure 1).

### Data processing

Figure 1 summarizes all of the data processing steps. The entire analysis was performed using QIIME 2 (Version 2021.2) (32). Paired-end reads were merged using VSEARCH with default parameters (33). The total number of sequencing reads was 434 900 445. The sequencing reads were quality filtered as follows; reads were truncated at any site containing >3 consecutive low-quality base (Phred score < 4), and the minimum fraction of consecutive high-quality bases to be retained was set to 75% of the length of the input sequence with no uncalled bases (Ns) (34). The total number of sequencing reads after the quality filtering was 432 039 098. The Deblur was used for denoising and chimera removal to obtain amplicon sequence variants (ASVs) using a trim length of 250 bases (29,35). The resulting ASVs from all samples were combined into a BIOM table (36). After using the Deblur, a total of 81 701 877 reads were obtained from 2601 samples (34 projects), with an average of 31 412 reads per sample (a minimum of 2 reads and maximum of 205 611 reads). Samples with a minimum of 1000 reads were included after denoising and chimera removal, and a total of 2530 samples from 34 projects were available (the total number of sequencing reads was 81 669 682).

For diversity analyses, we normalized sequencing reads to 1000 reads by rarefying (37) and scaling with ranked subsampling (SRS) (38). Alpha diversity indices, including the observed ASVs and the Shannon index (39), were calculated from samples before normalization, after rarefying and after SRS (40). All ASVs were aligned with MAFFT (41) and were used to construct a phylogenetic tree with FastTree 2 (40,42). Using the phylogenetic tree, we calculated unweighted and weighted UniFrac distances to measure beta diversity after rarefying samples to 1000 reads (37,43–47). Principal coordinate analysis (PCoA) was performed based on the unweighted and weighted UniFrac distances (48,49), and PCoA plots were visualized with Emperor (50,51).

For taxonomic analysis, taxonomy was assigned to ASVs using the q2-feature-classifier classify-consensus-vsearch (33,40,52) against the EzBioCloud (53). All matches with an identity percentage of 0.97 or higher were kept. We only used bacterial 16S rRNA gene sequences from the EzBioCloud. Multi-layered pie charts representing the taxonomic composition were visualized with Krona (54), and network graphs representing the associations between bacteria and hosts were visualized with Flourish (https://flourish.studio/).

### Web implementation

Data were stored in a MySQL database (https://www.mysql.com/). The backend was implemented in Java using Spring Boot (https://spring.io/projects/spring-boot). The user interface was implemented using CSS, HTML and JavaScript with Bootstrap (https://getbootstrap.com/), jQuery (https://jquery.com/) and Thymeleaf (https://www.thymeleaf.org/). DataTables (https://datatables.net/) and Plotly.js (https://plotly.com/javascript/) were used for data visualization. The ‘Visualization’ page was made by referring to the format of *Peryton* (55). We tested AMDB on Google Chrome, Microsoft Edge and Mozilla Firefox to provide a robust service. In addition, we made AMDB accessible and legible on phone and tablet screens.

## RESULTS

### Database statistics

**Figure 2.**
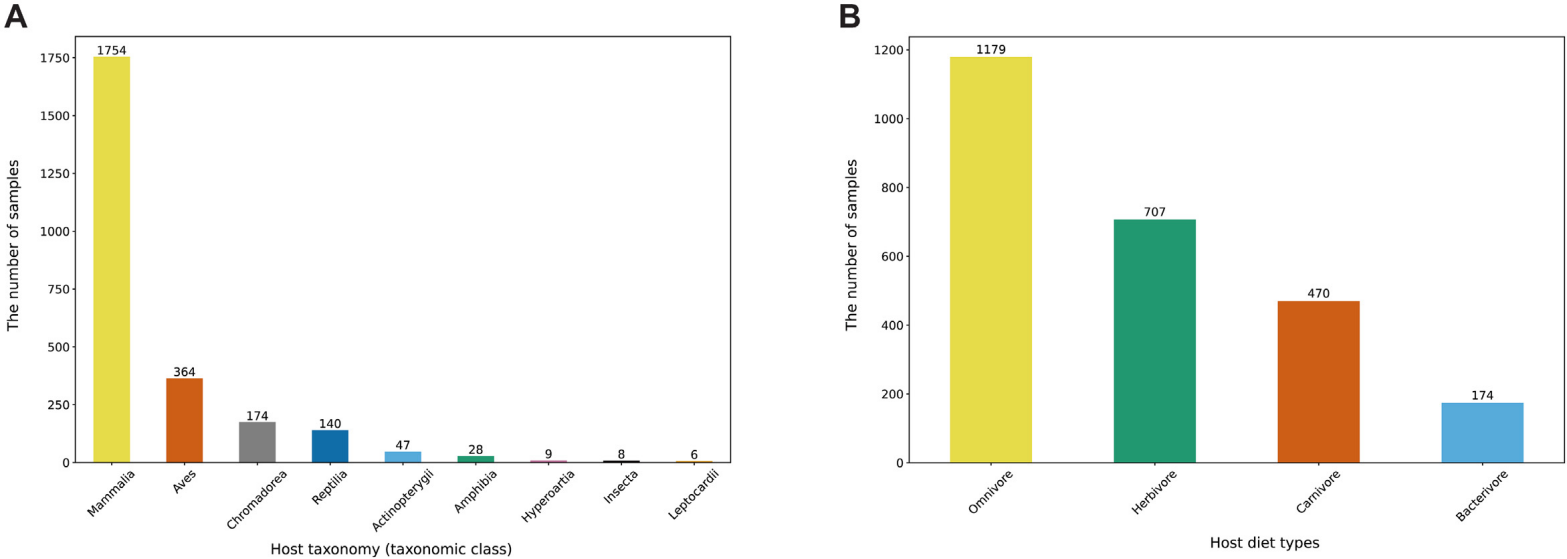
Bar plots showing the distribution of samples. (**A**) The number of samples is represented according to the host taxonomic classes. (**B**) The number of samples is represented according to the host diet types. Values are shown on the head of each bar. The bars are sorted in descending order by the values.

Table 1 summarizes the statistics of AMDB. AMDB contains 2530 samples from 34 projects. A total of 139 375 ASVs were identified, corresponding to 81 669 682 reads. In the taxonomic analysis, 84.94% (69 367 504) reads were assigned to bacterial taxa, covering a total of 10 478 taxa. The total number of hosts in AMDB was 467 animal species, representing nine taxonomic classes (namely, ‘Mammalia’, ‘Aves’, ‘Chromadorea’, ‘Reptilia’, ‘Actinopterygii’, ‘Amphibia’, ‘Hyperoartia’, ‘Insecta’ and ‘Leptocardii’) and four trophic groups (namely, ‘Omnivore’, ‘Herbivore’, ‘Carnivore’ and ‘Bacterivore’). The most abundant host taxonomic class was ‘Mammalia,’ which represented 69.33% (1754) of the samples, followed by ‘Aves’ and ‘Chromadorea’ which represented 14.39% (364) and 6.88% (174), respectively (Figure 2A). The most abundant trophic group was ‘Omnivore,’ which represented 46.60% (1179) of the samples, followed by ‘Herbivore’ and ‘Carnivore’ which represented 27.94% (707) and 18.58% (470) respectively (Figure 2B).

**Table 1.** Data summary of AMDB

Variable		N
**Samples**		2530
**Projects**		34
**Features (ASVs)**		139 375
**Sequencing reads**		81 669 682
**Bacterial taxa**		
	Phylum	44
	Class	115
	Order	280
	Family	687
	Genus	2828
	Species	6524
	**Total**	10 478
**Host taxonomy**		
	Class	9
	Order	63
	Family	180
	Genus	369
	Species	467
**Host diet types**		4

ASV: amplicon sequence variant

### Database content and usage

AMDB can be divided into four main parts, namely ‘Taxa’, ‘Samples’, ‘Projects/Hosts’ and ‘Visualization’. ‘Taxa’ shows samples enriched with the bacterial taxon of interest. ‘Samples’ provides the gut microbiota composition of the sample of interest. ‘Projects’ and ‘Hosts’ give users summary information on the project and the host, respectively. ‘Visualization’ visually presents valuable information related to the relationship between the host and the gut microbiota.

‘Taxa’ allows users to search for the taxon of interest (Supplementary Figure S1). ‘Taxa’ provides taxon information, including taxonomy and taxonomic rank. It also supports linking with the EzBioCloud (53), allowing for direct access to relevant information about the taxon. A list of the samples is provided, sorted according to the relative abundance of the taxon occurring within each sample. The AMDB allows the user to determine the relative abundance of the taxon based on host taxonomic ranks and host diet types using box plots. Each group name in the plot is followed by a frequency of occurrence, calculated as the number of samples containing the taxon divided by the total number of samples in the group. The viewing area of the plot can be adjusted by zooming in (dragging) or zooming out (double-clicking on the plot). Users can also click the camera icon to download the plot as a portable network graphics (.png) file.

‘Samples’ allows users to determine the gut microbiota composition of the sample of interest (Supplementary Figure S2). ‘Samples’ provides sample information, including the sample name, sampling site, accessions in the NCBI and information about the host and the respective analysis. Calculated alpha diversity indices are also displayed. A list of ASVs that make up the sample is provided. Taxonomic composition is displayed in a table and a multi-layered pie chart. The multi-layered pie chart has zooming capabilities, providing a more detailed view (double clicking on the node allows for zooming in, while zooming out is achieved by clicking on the summary pie charts present on the right-hand side of the chart). The ASV list and the taxonomic composition can be downloaded as comma-separated values (.csv) files.

Users are able to get summary information about a project and a host using ‘Projects’ (Supplementary Figure S3A) and ‘Hosts’ (Supplementary Figure S3B), respectively. ‘Projects’ provides information about the related paper, and ‘Host’ displays the taxonomy and diet type of the host. A complete list of samples related to both the project and the host is provided. Additionally, the alpha diversity indices from the samples are displayed as box plots. The average taxonomic composition from the samples is displayed in a table and a multi-layered pie chart. The sample list and the average taxonomic composition can be downloaded as comma-separated values (.csv) files.

**Figure 3.**
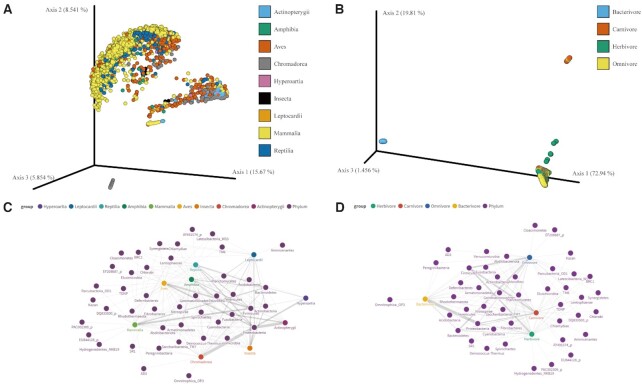
AMDB visualizations. (**A**) PCoA plot shows variation in phylogenetic structure among 2530 samples based on unweighted UniFrac distances. Each point represents an individual sample, and samples are colored by the host taxonomic class. (**B**) PCoA plot shows variations in phylogenetic structure among 2530 samples based on weighted UniFrac distances. Each point represents an individual sample, and samples are colored by the host diet. (**C**) Associations between bacteria and host taxonomic classes are shown as a network graph. Each node represents an individual taxon (the phylum level) or a host taxonomic class. (**D**) Associations between bacteria and host diet types are shown as a network graph. Each node represents an individual taxon (the phylum level) or a host diet type. For network graphs, the line width is proportional to the average relative abundance of each taxon.

AMDB provides interactive visualizations, including PCoA plots and network graphs, to effectively describe the relationship between the samples and the respective gut microbiota on the ‘Visualization’ page. Via PCoA plots, users can assess the variations in phylogenetic structure among the samples based on unweighted UniFrac distances (Figure 3A) and weighted UniFrac distances (Figure 3B). Each point in the plot represents one sample and can be colored-coded depending on the users’ choice. The plot is a draggable and zoomable 3D object, allowing users to view the plot from different perspectives. The plots can be downloaded as portable network graphics (.png) files, scalable vector graphics (.svg) files or QIIME 2 visualizations (.qzv) files (32). Network graphs show associations between the gut microbiota and host characteristics, including taxonomy (Figure 3C) and diet types (Figure 3D). Nodes in the network graphs represent taxa and host characteristics. The nodes can be moved and filtered out or in according to the users’ choice. Clicking on each node brings up a pop-up box with a link to ‘Taxa’ in a new tab. The line width of the network graph is relative to the average relative abundance of each taxon.

### Other functionalities

To better guide users, the ‘Help’ page provides an overview of AMDB with simple examples. Users can also propose candidate data for AMDB using the submission form on the ‘Contact’ page. Our team will manually check new user-submitted information, and AMDB will be updated on an ongoing basis.

## DISCUSSION

AMDB is a database for exploring the gut microbiota of various animal species. AMDB provides a search capability for the various components related to gut microbiota. For example, one may be interested in *Bilophila wadsworthia*, which is known to be related to animal-based diets in humans (56). The samples rich in this taxon can be identified in the search result of AMDB. Additionally, AMDB allows users to search for the sample based on metadata, including host taxonomy and diet types. The work from Youngblut *et al.* identified that hosts from the same species showed similar relative abundances of microbial phyla (7). This can be confirmed by comparing the microbial taxonomic compositions of samples taken from the same species. In addition, AMDB provides summary information about related projects and hosts. Users can thus compare the mouse information held within AMDB to the core microbiota of the mouse gut identified in multiple studies (57–59). Interactive visualizations are also available in AMDB. Host phylogeny and diet can explain variations in the gut microbiota (7), which can be confirmed using a PCoA plot within AMDB. The phylum Proteobacteria was identified as the dominant phylum in the samples from Actinopterygii (60), which can be identified using the network graph.

The number of available amplicon data in the NCBI SRA is continually increasing. AMDB will also be continuously updated to add additional data related to new and existing animal species. We will include new data collected by our team, as well as data based on the user-submitted information after manual curation.

Investigations into the relationship between gut microbiota and the host is a rapidly growing area of research (61). AMDB is the first database enabling easier exploration of this relationship. AMDB comprehensively addresses the taxonomic composition of animal gut microbiota with manually curated metadata, thus assisting in providing a better understanding of the gut microbiota of animals.

## DATA AVAILABILITY

AMDB is freely available at http://leb.snu.ac.kr/amdb.

## Supplementary Material

gkab1009_Supplemental_File

## Notes

Present address: Jongsik Chun, Department of Biological Sciences, Seoul National University, Seoul 08826, Korea.
