# Supplementary material for: AMDB: a database of animal gut microbial communities with manually curated metadata
Source: Nucleic Acids Res. 2021 Nov 8;50(D1):D729–35. doi: 10.1093/nar/gkab1009 (PMC8728277; doi:10.1093/nar/gkab1009)
Supplement: gkab1009_Supplemental_File [file gkab1009_supplemental_file.pdf]

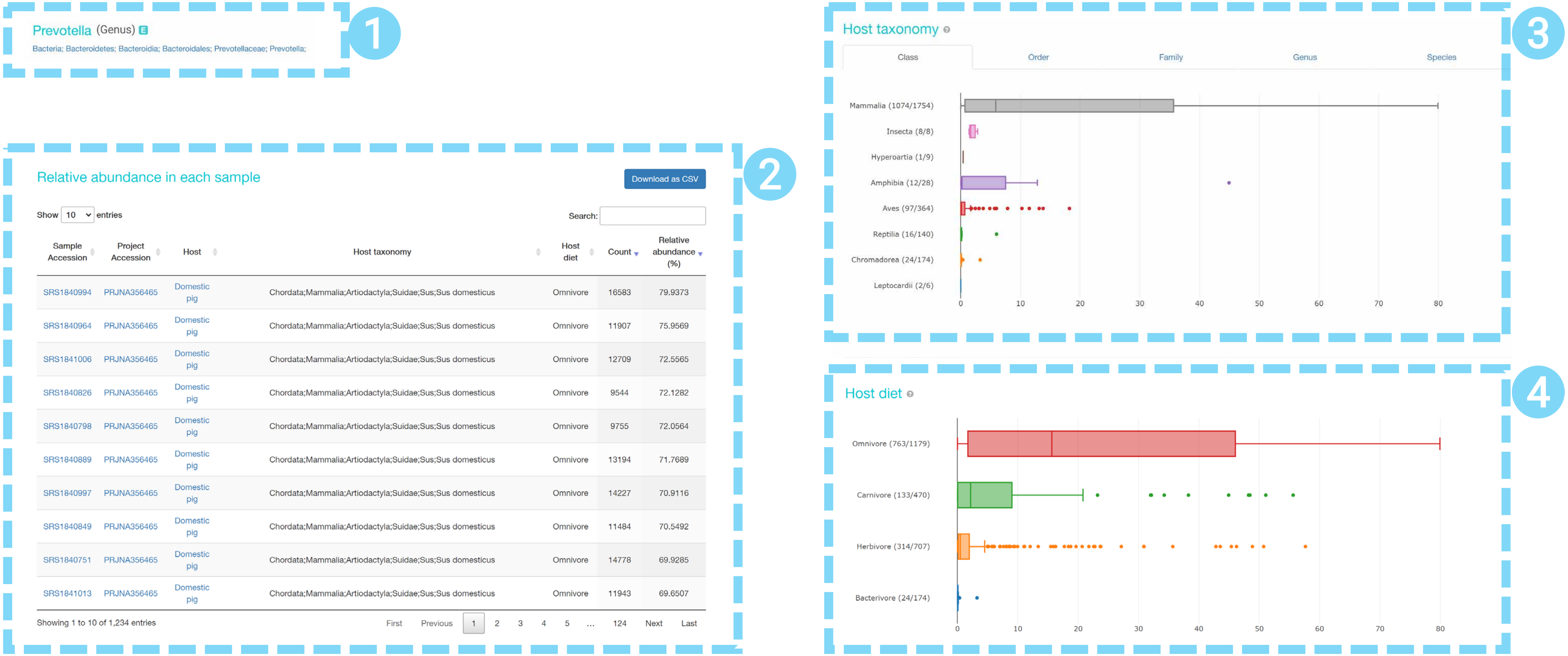

Supplementary Figure S1. The interface of 'Taxa.' Users can search for the taxon of interest (e.g., *Prevotella*). Taxon information is first provided in the results (1). It includes the taxonomy and rank of the taxon. A link to the EzBioCloud is also provided for more information on the taxon. Samples containing the taxon are shown (2). The samples are ordered based on the relative abundance of the taxon within the samples. Box plots allow the users to determine the relative abundance of the taxon based on the host taxonomic ranks (3) and host diet type (4). Each group name in the plot is followed by a frequency of occurrence, calculated as the number of samples containing the taxon divided by the total number of samples in the group. The groups are sorted according to the median.

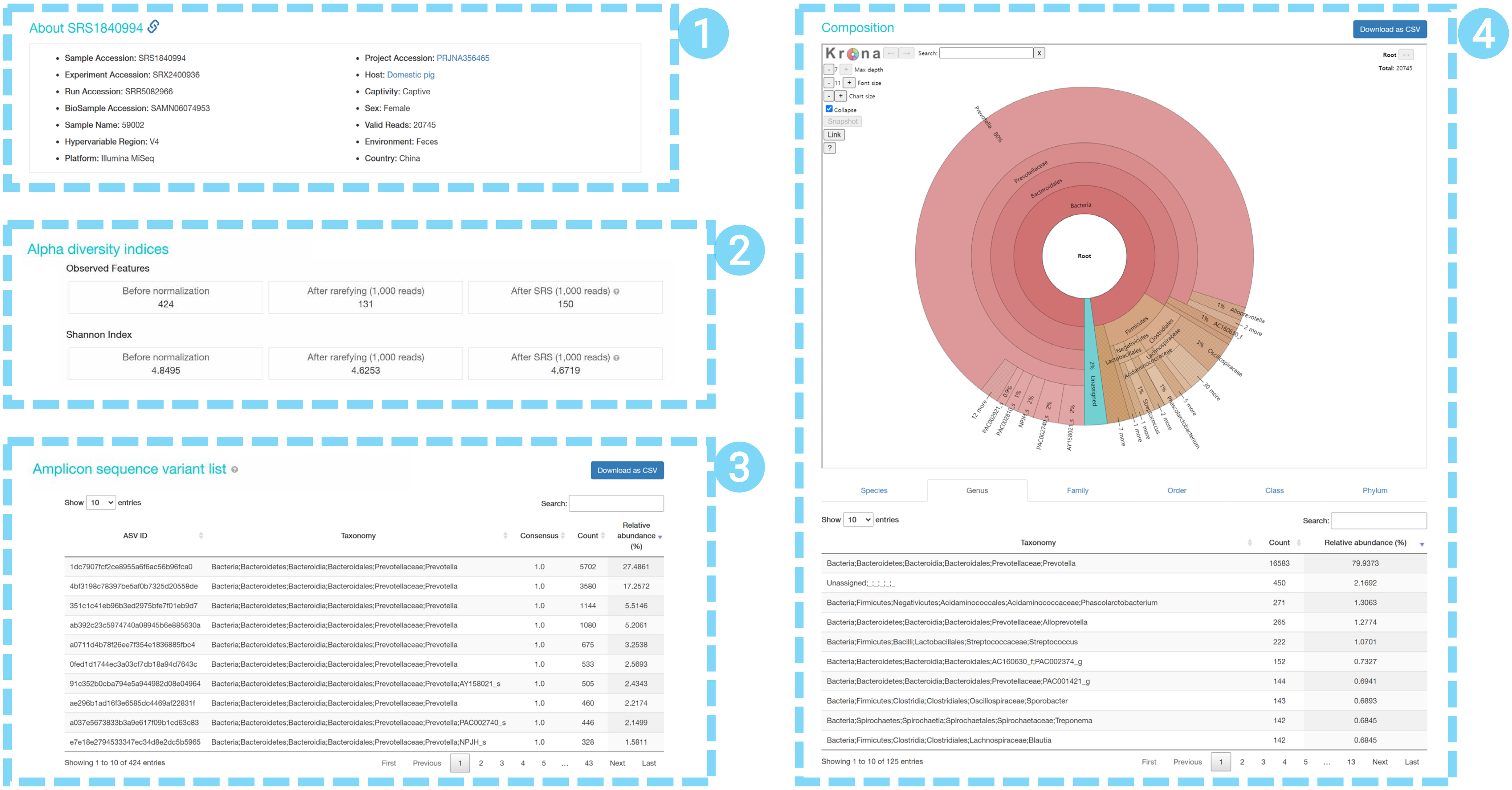

Supplementary Figure S2. The interface of 'Samples.' Users can determine the gut microbiota composition of the sample of interest. Sample information is first given in the results (1). It includes the sample name, sampling site, accessions in the NCBI and information about hosts and analyses. A link to the NCBI is also provided. The alpha diversity indices are displayed (2). A list of amplicon sequence variants (ASVs) that compose the sample is provided (3). The composition is plotted and tabulated using the ASV lists (4). The table contents can be downloaded as comma-separated values (.csv) files through the download button.

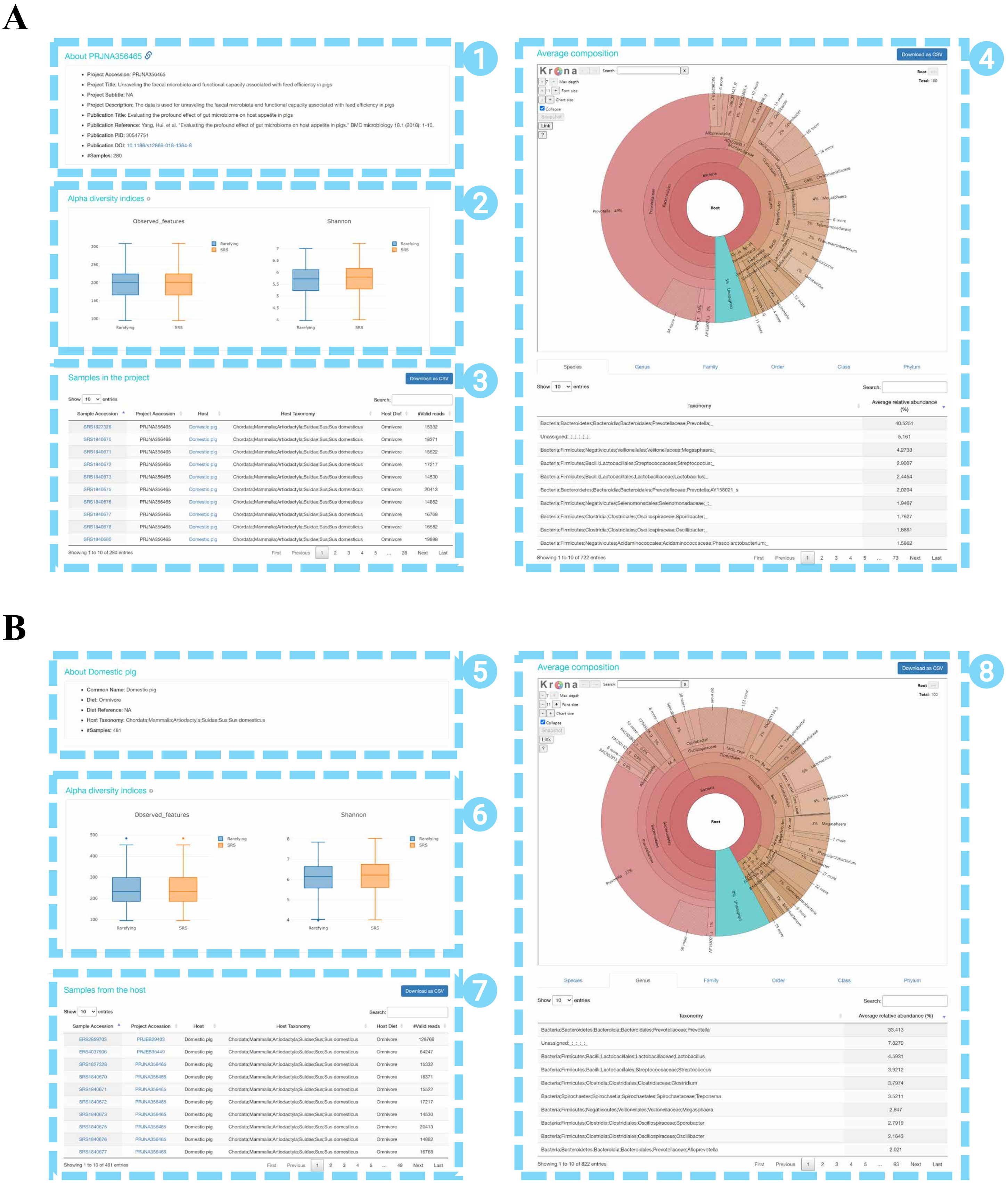

Supplementary Figure S3. Interfaces of 'Projects' and 'Hosts.' (A) In 'Projects,' users can get summary information about the project. Project information is first given in the results (1). It includes information about the related paper. A link to the NCBI is also provided. The alpha diversity indices from the samples included in the project are plotted (2). A complete list of samples included in the project is provided (3). The average composition is plotted and tabulated (4). (B) In 'Hosts,' users can get summary information about the host. Host information is first given in the results (5). It includes the taxonomy and diet type of the host. The alpha diversity indices from the samples related to the host are plotted (6). A complete list of samples related to the host is provided (7). The average composition is plotted and tabulated (8). The table contents can be downloaded as comma-separated values (.csv) files through the download button.
